# Quantifying the fractal complexity of nutrient transport channels in Escherichia coli biofilms under varying cell shape and growth environment

**DOI:** 10.1099/mic.0.001511

**Published:** 2024-11-05

**Authors:** Beatrice Bottura, Liam Rooney, Morgan Feeney, Paul A. Hoskisson, Gail McConnell

**Affiliations:** 1 Department of Physics, SUPA, University of Strathclyde, G4 0NG, Glasgow, UK; 2 Strathclyde Institute of Pharmacy and Biomedical Sciences, University of Strathclyde, G4 0RE, Glasgow, UK

**Keywords:** Biofilms, Image analysis, Microscopy

## Abstract

Recent mesoscopic characterization of nutrient-transporting channels in *Escherichia coli* has allowed the identification and measurement of individual channels in whole mature colony biofilms. However, their complexity under different physiological and environmental conditions remains unknown. Analysis of confocal micrographs of colony biofilms formed by cell shape mutants of *E. coli* shows that channels have high fractal complexity, regardless of cell phenotype or growth medium. In particular, colony biofilms formed by the mutant strain Δ*ompR*, which has a wide-cell phenotype, have a higher fractal dimension when grown on rich medium than when grown on minimal medium, with channel complexity affected by glucose and agar concentrations in the medium. Osmotic stress leads to a dramatic reduction in the Δ*ompR* cell size but has a limited effect on channel morphology. This work shows that fractal image analysis is a powerful tool to quantify the effect of phenotypic mutations and growth environment on the morphological complexity of internal *E. coli* biofilm structures. If applied to a wider range of mutant strains, this approach could help elucidate the genetic determinants of channel formation in *E. coli* colony biofilms.

## Introduction

The formation of spatial patterns is ubiquitous in biological systems, where discrete entities come together to form complex structures in a process called ‘morphogenesis’ [1]. Bacterial populations are no exception to this phenomenon: bacterial biofilms exhibit a variety of internal patterns, from surface and 3D features to complex fractal shapes [2]. Fractal geometry has previously been proposed as a tool for the investigation of microbial growth patterns [3], and it has since been employed to quantify biofilm morphology from microscopy images [4,6], describe colony morphogenesis in Gram-negative rod-shaped bacteria [7] and analyse nutrient-limited growth patterns [5,8].

In *Escherichia coli*, fractal patterns mostly exist within cellular aggregates of co-cultured isogenic strains expressing different fluorescent makers [9,10]. Fractal boundaries are formed during uniaxial cell growth and division and can be a result of local instabilities [11]. Alternatively, fractal domains can be observed between mutant sectors arising from genetic differences in the population [12], where mutants with a fitness advantage grow faster and gain greater access to nutrients at the periphery of the colony [13]. Metabolic interactions between isogenic strains can lead to different patterns of self-organization from uniform radial expansion to the formation of dendritic niches at the colony edge [14]. Fractal boundaries have also been observed in * E. coli* co-cultures of cross-feeding strains, where the type of social interaction determines the level of spatial mixing between the two strains [15]. The degree of self-similarity across the boundaries, usually measured through the fractal dimension, depends on the properties of the constituent cells.

The network of nutrient-transporting channels in *E. coli* colony biofilms [16] also exhibits a complex 3D morphology. The spatial structure of these emergent channels bears a striking resemblance to the fractal boundary patterning described in multi-strain co-culture biofilms, although these channels are not occupied by cells. By measuring the width of individual channels at different locations within the biofilm, previous work showed that the channel architecture is affected by environmental growth conditions [17], but quantification of channel morphology at the whole-biofilm scale has not been performed to date.

In this study, we quantify the morphological complexity of *E. coli* nutrient-transporting channels using fractal analysis of confocal microscopy images of colony biofilms. We hypothesized that their fractal nature would be affected by the shape of the constituent cells because channel formation is an emergent property of biofilm growth. To test this hypothesis, we selected three *E. coli*-mutant strains with altered cell shape phenotypes for morphological analysis. We show that the internal patterns formed by nutrient-transporting channels in *E. coli* have a fractal complexity comparable to that of computer-generated fractal images and that this complexity varies depending on the genotype of the constituent strain, which controls cell shape phenotype, and by growth medium. We also report the specific case of Δ*ompR*, a wide-cell mutant whose colony biofilm morphological complexity is particularly affected by growth medium composition.

## Methods

### Strains and media

The bacterial strains used in this work (Table S1, available in the online Supplementary Material) were obtained from the Keio collection [18], a single-gene knockout library of all nonessential genes in the *E. coli* K-12 strain, BW25113 [19]. The mutants Δ*amiA*::kan, Δ*ompR*::kan and Δ*ydgD*::kan of the *E. coli* strain BW25113, referred to in the text as Δ*amiA*, Δ*ompR* and Δ*ydgD,* were selected for their modified cell phenotype, and their single-gene deletions were verified by PCR and DNA amplicon sequencing (Table S2). The strains were transformed by electroporation with the plasmid pAJR145, which is derived from the plasmid pACYC184 and encodes the constitutively expressed GFP transcriptional fusion *rpsM::gfp+* [20]. Prior to transformation, liquid cultures of each strain were made electrocompetent through three ice-cold 10% glycerol washes.

Liquid cultures were grown overnight in a 37 °C aerated incubator while shaking at 250 r.p.m. in Miller’s lysogeny broth (LB) [21] with the addition of 25 µg ml^−1^ chloramphenicol to maintain GFP fluorescence from the pAJR145 plasmid. *E. coli*-mutant strains obtained from the Keio collection were also grown with the addition of 50 µg ml^−1^ kanamycin. M9 minimal medium salts [21] were prepared as a 5× solution, then diluted to 1× with distilled deionized water and supplemented with 1 mM MgSO_4_·7H_2_O, 0.2% (w/v) glucose and 0.00005% (w/v) thiamine. Solid substrates were prepared by adding 20 g l^−1^ of agar and colony biofilms were grown in a 37 °C static aerated incubator.

Solid growth substrates were also prepared with different nutritional profiles and agar concentrations to quantify the resulting change in the internal morphology of Δ*ompR* colony biofilms. No-salt agar substrates were prepared without NaCl, and soft agar substrates were prepared by reducing the amount of agar to 10 g l^−1^. d-Glucose was added to both LB and M9 media to final concentrations of 0.02% (w/v), 0.2% (w/v) or 0.5% (w/v).

### Growth characterization of bacterial strains in liquid growth media

Overnight cultures of BW25113 and Δ*amiA*, Δ*ompR* and Δ*ydgD* mutant strains were prepared in LB and diluted to a starting OD_600_ of 0.01 for growth curves in LB medium. For growth curves in M9 medium, overnight cultures were washed and resuspended in 1× M9 salts thrice and then diluted to a starting OD_600_ of 0.01. Three wells of a 96-well plate were then filled with 200 µl of diluted liquid cultures for each strain in both LB and M9 media. Wells containing sterile LB and M9 media were included as a ‘blank’, and the absorption values were subtracted from the growth curves for each measurement point. The plate was then loaded onto a pre-warmed (37 °C) Synergy HT plate reader (BioTek, USA), where the OD_600_ of the cultures was measured every 2 min for 16 h while the plate was shaking continuously at a medium speed.

The growth curves were plotted in Prism by exporting data from the Gen5 microplate software (BioTek), with the *y*-axis plotted on a logarithmic scale. Specific growth rates were calculated as the slope of the linear region of the semi-logarithmic plot using the equation,

$$\mu =\frac{2.303({log}_{10}N-{log}_{10}N_{0})}{\left(t-t_{0}\right)}$$

where *N*_0_ and *N* are the cell numbers at the beginning and at the end of the exponential growth phase, respectively, and correspond to OD readings, and *t*_0_ and *t* are the times at which the exponential growth phase starts and ends, respectively [22].

### Growth characterization of BW25113 and Δ*ompR* in high-osmolality medium

LB was prepared with iodixanol concentrations of 0, 1, 2, 5, 10 and 20% (v/v) by adding appropriate amounts of an OptiPrep 60% (w/v) iodixanol stock solution (Sigma-Aldrich, USA). Ten wells of a 96-well plate were then filled with 200 µl of medium with each iodixanol concentration (corresponding to five biological replicates for each strain). Overnight cultures of BW25113 and the Δ*ompR* mutant strain were prepared in LB as described above and were diluted to a starting OD_600_ of 0.01. The plate was then loaded onto a pre-warmed (37 °C) Synergy HT plate reader (BioTek), where the OD_600_ of the cultures was measured every 15 min for 24 h while the plate was shaking continuously at a medium speed. Specific growth rates were calculated as described above.

To compare cell shape phenotype before and after growth in a high-osmolality medium, BW25113 and Δ*ompR* were first grown as liquid cultures overnight in LB. They were then diluted 1 : 100 in both LB and LB with 20% (v/v) iodixanol and were incubated further in a 37 °C aerated incubator while shaking at 250 r.p.m. until mid-exponential growth phase, before imaging using phase-contrast microscopy as described below. Solid LB medium plates were prepared with iodixanol concentrations of 1, 10 and 20% (v/v) and used as substrates for the growth of BW25113 and Δ*ompR* colony biofilms.

### Single-cell phase-contrast microscopy

For single-cell imaging, overnight cultures of each strain in LB were diluted 1 : 100 and incubated for 2 h until they reached the mid-exponential growth phase (OD_600_ = 0.4–0.6). Imaging slides were prepared by sandwiching 1 ml of molten 1% agarose between two microscope slides and letting it solidify at room temperature. After removing the top slide, 10 µl of liquid culture was spotted onto the solidified agarose pads, and a coverslip was added prior to imaging. Single-cell phase-contrast images were acquired for both non-fluorescent strains (BW25113, BW25113 Δ*amiA*::kan, BW25113 Δ*ompR*::kan and BW25113 Δ*ydgD*::kan) and fluorescent strains (BW25113/pAJR145, BW25113 Δ*amiA*::kan/pAJR145 and BW25113 Δ*ompR*::kan/pAJR145, BW25113 Δ*ydgD*::kan/pAJR145) to ensure that the addition of chloramphenicol required for the maintenance of the pAJR145 plasmid did not affect the cell phenotype of each strain.

Single-cell imaging was carried out using an Eclipse E600 upright widefield microscope (Nikon, Japan) in phase-contrast mode equipped with a 100×/1.30 DLL oil immersion lens (Nikon). Illumination was provided by a halogen lamp, and the image was detected using a Hamamatsu ORCA-100 digital camera (Hamamatsu, Japan).

### Confocal microscopy of colony biofilms

The GFP-expressing strains BW25113/pAJR145, BW25113 Δ*amiA*::kan/pAJR145, BW25113 Δ*ompR*::kan/pAJR145 and BW25113 Δ*ydgD*::kan/pAJR145 were grown into mature colony biofilms on agar substrates in a sterile 3D-printed plastic specimen holder, as described previously [16]. Colony biofilms were grown for 24 h when using LB medium and for 48 h when using M9 medium. This difference in growth is due to the doubling time of *E. coli* in M9 medium being half that of the doubling time in LB medium [23].

Mature colony biofilm images were acquired on an Olympus IX81 microscope coupled to a FluoView FV1000 confocal laser scanning unit (Olympus, Japan). Fluorescence from GFP was excited using a 488 nm argon laser (GLG3135, Showa Optronics, Japan) and was detected by a photomultiplier tube (PMT) with a spectral detection window set between wavelengths of 510 and 560 nm. Samples were imaged using a 10×/0.4 air objective lens for resolving intra-colony channels.

Three-dimensional *z*-stacks of colony biofilms grown on LB and M9 media were acquired with a slice spacing of 5 µm for Nyquist sampling in the axial dimension. Five different colony biofilms were imaged for each condition.

### Image analysis

Phase-contrast images of both fluorescent and non-fluorescent cells were first preprocessed in FIJI using the background subtraction tool with a rolling ball radius of 15 pixels, with the ‘light background’ option selected. The images were then analysed using the FIJI plugin MicrobeJ [24] to obtain cell length and width measurements. Segmentation was performed using MicrobeJ with the default settings for a bright background and the ‘medial axis’ mode of detection. The following changes to the default parameter ranges were made: area (1–max) µm^2^, width (0–max) µm with variation (0–0.2), sinuosity (1–1.2), angularity (0–0.5) rad and solidity (0.9–max). The default ‘advanced’ parameters were changed to have an area cut-off of 1000 and a count cut-off of 250. The options ‘exclude on edges’ and ‘shape descriptors’ were selected. Phase-contrast images were analysed (*n* = 10 for each strain), with a total number of analysed cells between 202 and 665 (non-fluorescent cells) and between 90 and 524 (fluorescent cells).

Colony biofilm image *z*-stacks were displayed using FIJI Temporal Color Code Hyperstacks and colour coded by depth using the ‘fire’ lookup table. Biofilm images were contrast-adjusted where needed for presentation purposes, using contrast-limited adaptive histogram equalization [25] available in FIJI with block size 60, maximum slope 3 and 256 histogram bins. Regions of interest in biofilm images were despeckled using the ‘Process/Noise/Despeckle’ function in FIJI.

### Fractal complexity quantification

For fractal pattern analysis of colony biofilms, *z*-stacks were converted into 8-bit images (corresponding to greyscale values between 0 and 256). No contrast adjustment was performed on the images prior to analysis, ensuring that the analysed greyscale values faithfully represented the GFP signal from bacterial cells.

Fractal complexity was quantified using the FIJI plugin ComsystanJ (Complex Systems Analysis for ImageJ) [26], version 1.0.0 (https://github.com/comsystan/comsystanj)https://github.com/comsystan/comsystanj). The ‘2D image’ version of the plugin was chosen because it allowed the analysis of greyscale biofilm images through the relative differential box counting (RDBC) algorithm developed by Jin *et al*. [27], which uses a raster box scanning method. The 3D version of the algorithm, which only works in binary mode, was not able to capture the channel structures as accurately. Box-counting fractal dimension was calculated from hyperstacks of colony biofilm images, all of which had a size of 2048×2048 pixels, and the plugin was run with 12 boxes and 1–12 regressions.

For benchmarking of RDBC values, four sets of sample images were analysed using the 2D RDBC algorithm with the parameters described above. Three of the sample sets were generated as greyscale images through ComsystanJ using the ‘2D image Image Generator’ option, with image types ‘Constant’, ‘Fractal random shapes – Lines’ and ‘Random’. Constant images had a constant pixel intensity value (256) throughout each image. Line images were made of white (pixel intensity = 256) lines with varying thickness randomly intersecting on a dark (pixel intensity = 0) background. Fractal images were obtained from the ‘Wikimedia Commons’ open-source image repository [28,32]. These images were resized to 2048×2048 pixels and converted into 8-bit before analysis with ComsystanJ for consistency. Finally, in Random images, every pixel had a random intensity value (between 0 and 256).

### Statistical analysis

Statistical tests were carried out on Prism version 8.0.2 (GraphPad Software, USA). Cell measurements (length and width) of the mutant strains were compared to those of the parental strain BW25113 using a Kruskal–Wallis multiple comparison test, due to the non-normal distribution of the data. One-way ANOVA tests were used to compare box-counting dimensions of colony biofilms formed by different strains in the same media and also to compare box-counting dimensions of the same strain grown in different media.

In the main text and figure captions, values are presented as mean ± sd. In all plots, produced using Prism version 8.0.2, *P*-values are presented as follows: *P < 0.05, **P < 0.005, ***P < 0.0005 and ****P < 0.0001, with specific *P*-values written in the figure captions.

## Results

### Cell shape and growth medium affect the morphology of colony biofilms

The single knockout mutants Δ*amiA*::kan, Δ*ompR*::kan and Δ*ydgD*::kan of the *E. coli* strain BW25113, hereby referred to as Δ*amiA*, Δ*ompR* and Δ*ydgD*, were chosen from the Keio collection for their altered cell shape phenotype (long, wide and wide, respectively). The length and width of individual segmented cells were calculated for each strain from phase-contrast microscopy images (Fig. S1) and compared to the measurements of the isogenic parental strain BW25113 for quantitative phenotypic analysis. Δ*amiA* cells were on average 34% longer than the parental strain, whereas Δ*ompR* and Δ*ydgD* cells were on average 48 and 23% wider than the parental strain, respectively.

**Fig. 1.**
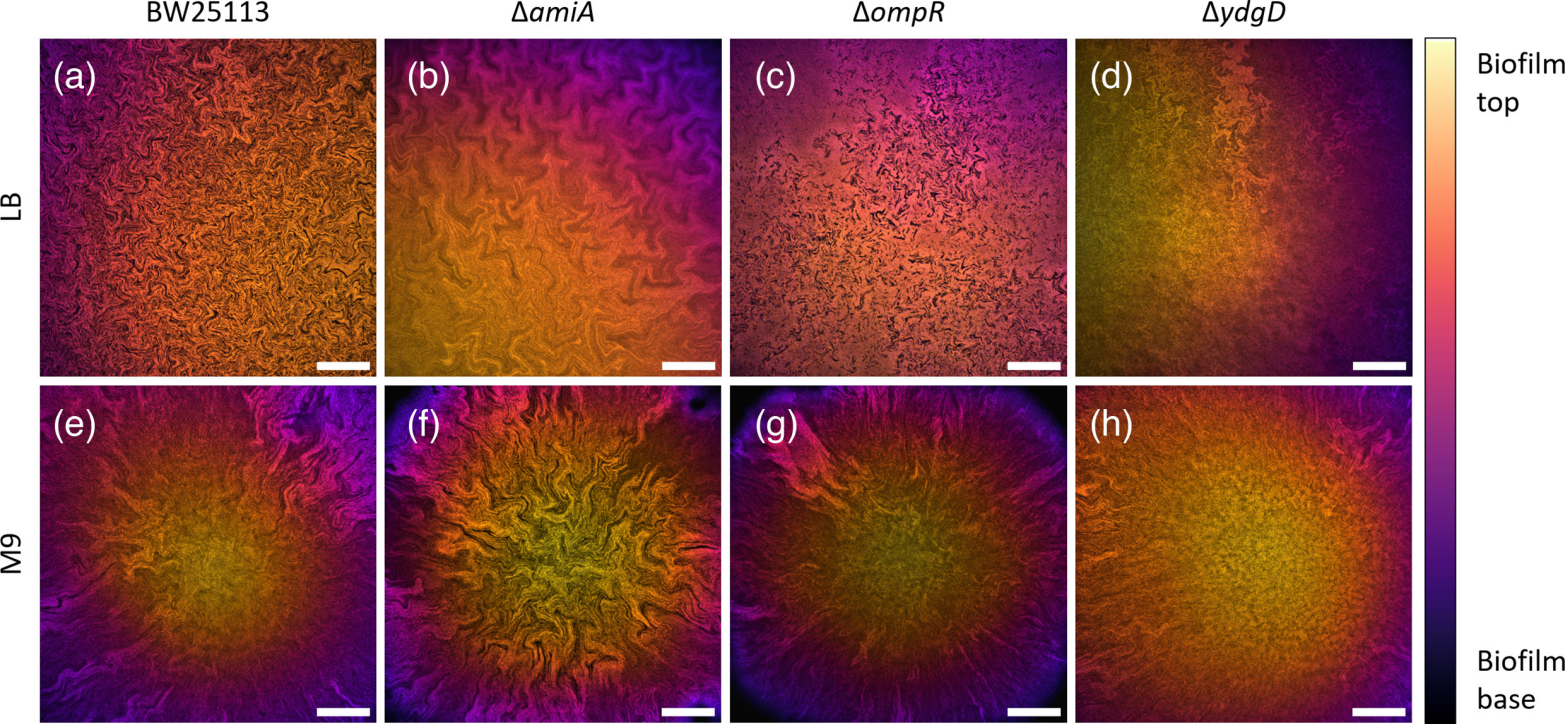
Representative confocal microscopy images showing the morphology of colony biofilms formed by the strains BW25113, Δ*amiA*, Δ*ompR* and Δ*ydgD* when growing on rich (LB) or minimal (M9) solid media. Images are projections of *z*-stacks acquired with 5 µm slice spacing, colour-coded by depth. Stacks were acquired over a total thickness of 160 µm (a), 145 µm (b), 130 µm (c), 165 µm (d), 165 µm (e), 220 µm (f), 180 µm (g) and 190 µm (h). Scale bars: 200 µm.

After single-cell phenotypic characterization, the three mutant strains were grown into mature colony biofilms on both LB (rich) and M9/glucose (minimal) solid growth media (Fig. 1), and their physiological state in both growth conditions was monitored by growth curve measurements (Fig. S2). Confocal microscopy of these biofilms revealed a complex network of intra-colony channels, as previously reported for *E. coli* JM105 [16,17]. Biofilms formed on minimal medium exhibited sectoring, with large areas of low fluorescence intensity across the biofilm volume, and channels appeared less morphologically complex than in their rich medium counterparts. This was particularly evident in the Δ*ompR* strain, where channels expand radially outwards in approximately straight lines, reminiscent of strain boundaries observed between isogenic domains in colony biofilms formed by spherical mutants of *E. coli* [11]. On rich medium, channels had more complex arrangements – in particular, channels formed by the Δ*amiA* strain were made up of long (≥10 µm) segments, consistent with the long-cell phenotype of the constituent cells. Channels formed by the Δ*ompR* mutant strain, on the other hand, appeared fragmented, likely owing to the constituent cells’ irregular morphology. Furthermore, despite both Δ*ompR* and Δ*ydgD* strains having a wide-cell phenotype, their biofilms had different internal morphologies, especially on rich medium. Here, Δ*ompR* formed darker channels, which were clearly distinguishable from the surrounding cells within the biofilm. On minimal medium, the central region of Δ*ydgD* biofilms appeared devoid of channels, whereas the edges showed radially expanding channels highly similar to those observed at the edges of Δ*ompR* biofilms.

### Internal channel patterns in *E. coli* colony biofilms are fractal

The fractal complexity of internal colony biofilm patterns was quantified using the metric of RDBC dimension, which was calculated using the open-source plugin ComsystanJ on FIJI [26]. The RDBC method is based on the box-counting algorithm, by which the image containing the fractal pattern is divided into a finite number of boxes with equal size, and the number of boxes containing the pattern outline is counted in relationship to the box size [33]. The RDBC dimension is a measure of fractal complexity calculated iteratively through the algorithm by varying the box size.

**Fig. 2.**
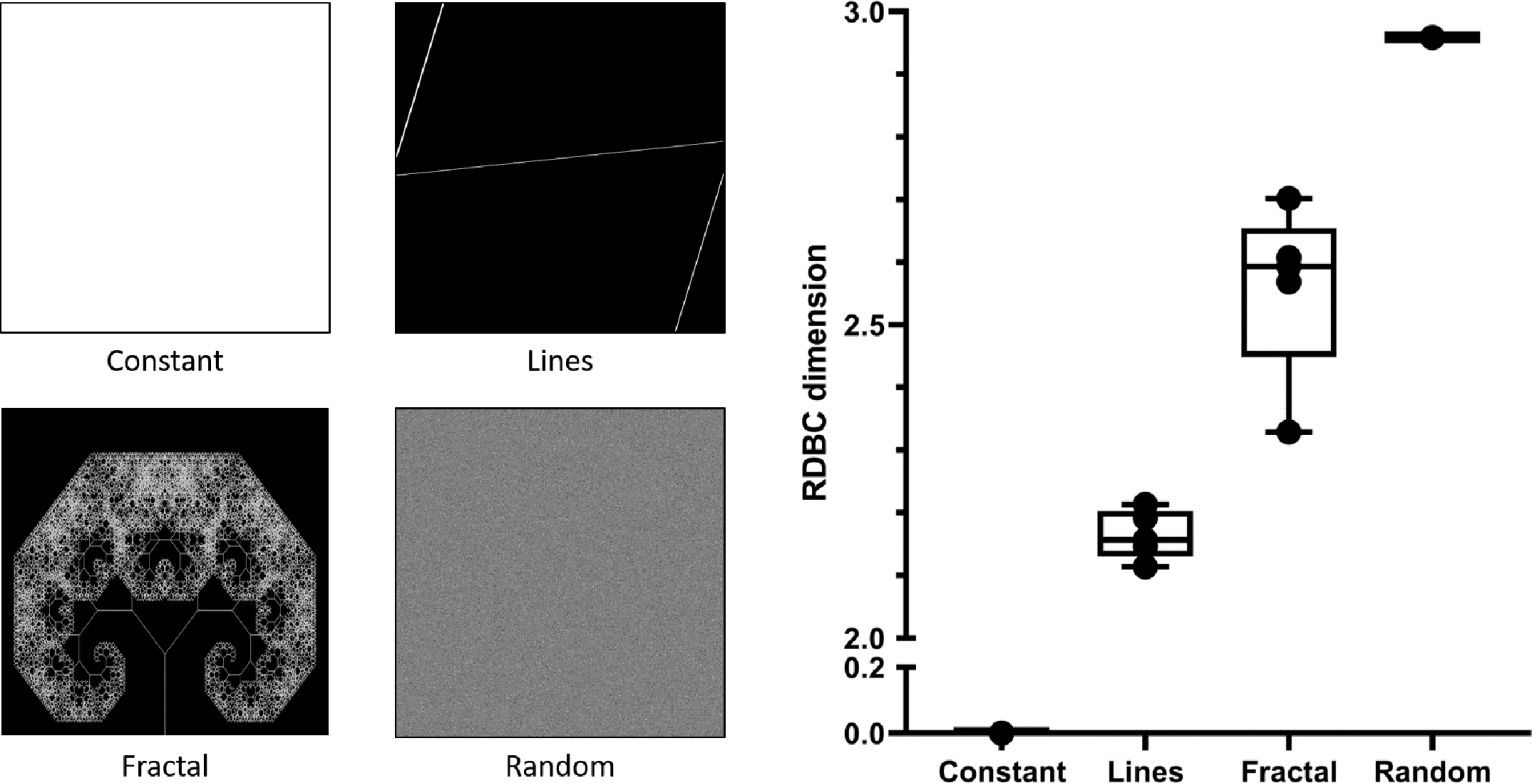
Representative images from the sets used for the benchmarking of RDBC calculations (right panel). Each set consists of five images, whose fractal complexity is calculated using ComsystanJ. The constant, lines and random images were generated in ComsystanJ, whereas the fractal images were obtained from open-source image repositories. Increasing complexity in the image sets is reflected by an increase in relative differential box-counting dimension, which ranges between 2.329 ± 0.041 and 2.702 ± 0.047 for fractal images.

The plugin was first used on four sets of computer-generated images with increasing spatial complexity (as described in the Methods section) to obtain a correlation between visual complexity and RDBC dimension (Fig. 2). Constant images had an RDBC dimension of 0 due to the lack of spatial features, whereas the RDBC dimension of images comprising straight lines ranged between 2.114 ± 0.032 and 2.213 ± 0.043 (mean ± sd). The RDBC dimension for computer-generated fractal images was calculated to be between 2.329 ± 0.041 and 2.702 ± 0.047, and images of randomized features had an RDBC dimension equal to 2.959, close to the theoretically possible maximum of 3.000.

**Fig. 3.**
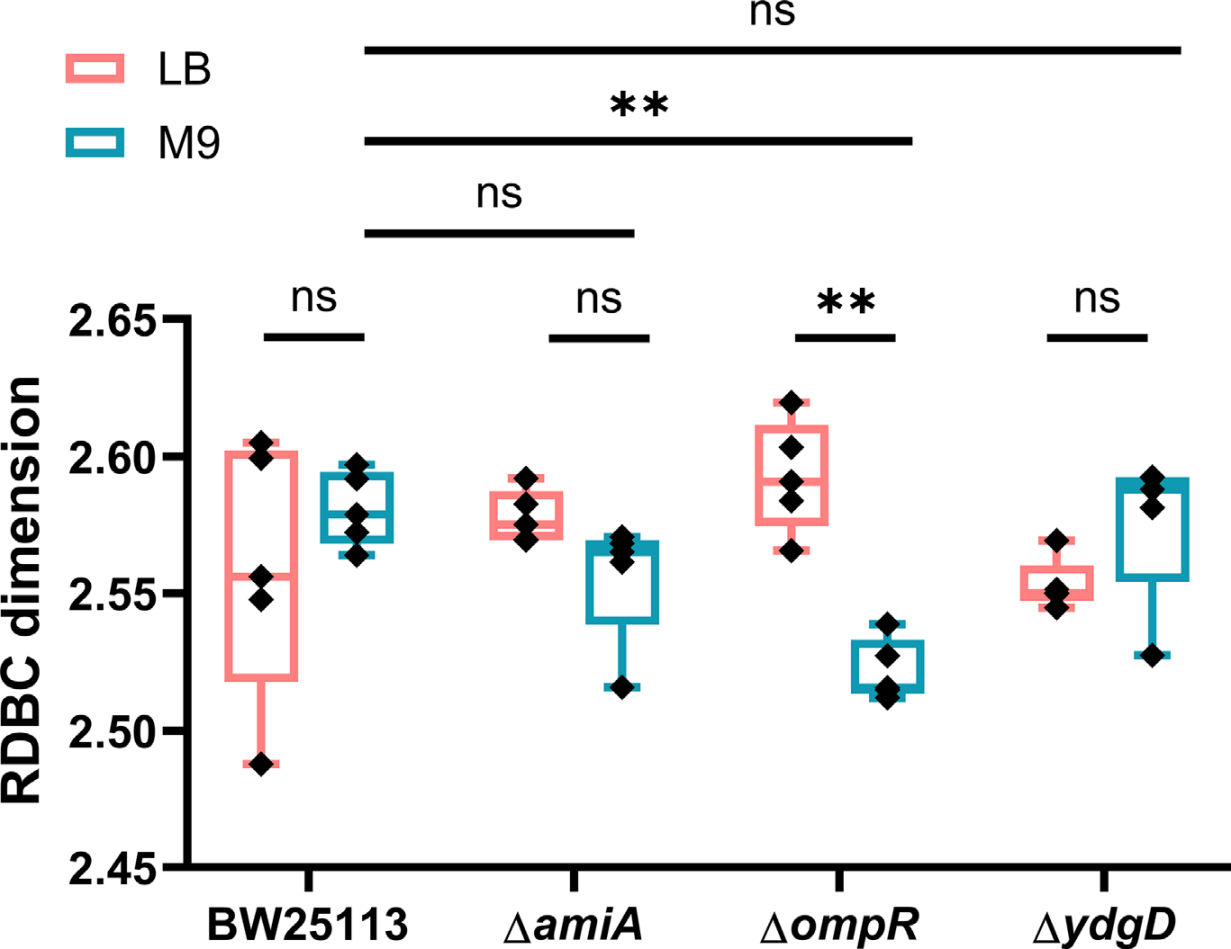
Box-counting dimension calculated from confocal micrographs of colony biofilms (*n* = 5 biofilms imaged for each condition). On minimal medium (M9), biofilms formed by the Δ*ompR* mutant strain are less morphologically complex than those formed by the parental strain (*P* = 0.0005, **). For the Δ*ompR* mutant strain, the fractal complexity is also strongly affected by the growth substrate composition, with biofilms grown on rich medium (LB) showing significantly more complexity than those grown on minimal medium (*P* = 0.0008, **). Average values are compared using one-way ANOVA tests.

After obtaining the benchmark values for fractal complexity, confocal microscopy images of colony biofilms were analysed using the same image analysis pipeline to investigate the effect of cell shape and growth medium on the complexity of internal biofilm patterns (Fig. 3). The RDBC dimension values obtained from colony biofilm images matched those calculated for the set of computer-generated fractal images, with a minimum of 2.488 and a maximum of 2.620. This confirmed that channel structures in *E. coli* colony biofilms were fractal and that they could be quantified using fractal geometry.

On rich medium, biofilms formed by the Δ*amiA* mutant strain showed increased fractal complexity (RDBC = 2.578 ± 0.009) when compared to biofilms formed by Δ*ydgD* (RDBC = 2.553 ± 0.009, *P* = 0.0277). This reflects the peculiar channel architecture exhibited by Δ*amiA* biofilms grown on LB medium (Fig. 1). On minimal medium, the parental strain BW25113 formed more complex biofilms (RDBC = 2.581 ± 0.014) than the Δ*ompR* mutant (RDBC = 2.522 ± 0.011, *P* = 0.0005). Biofilms formed by the mutant Δ*ydgD* were also less complex (RDBC = 2.576 ± 0.028) than those formed by Δ*ompR* (*P* = 0.0013) despite both strains having a wide cell shape phenotype. This is likely a result of phenotypical differences between the two strains at the single-cell level. As shown in Fig. S1, Δ*ompR* cells exhibit an irregular, bulgy phenotype compared to the wide rods formed by Δ*ydgD*.

As shown in the image data presented in Fig. 1, growth on minimal media was objectively correlated with large, dark sectors within the colony biofilm, which we hypothesized would result in a lower fractal complexity. However, after quantifying channel morphology through the RDBC dimension and comparing each strain grown on the two different growth media, we observed no statistical difference. An important exception to this observation was the Δ*ompR* mutant strain, for which growth in minimal medium was associated with significantly lower morphological complexity than growth in rich medium (*P* = 0.0008). This finding is investigated in more detail in the following sections.

### Growth medium composition affects the complexity of Δ*ompR* colony biofilms

**Fig. 4.**
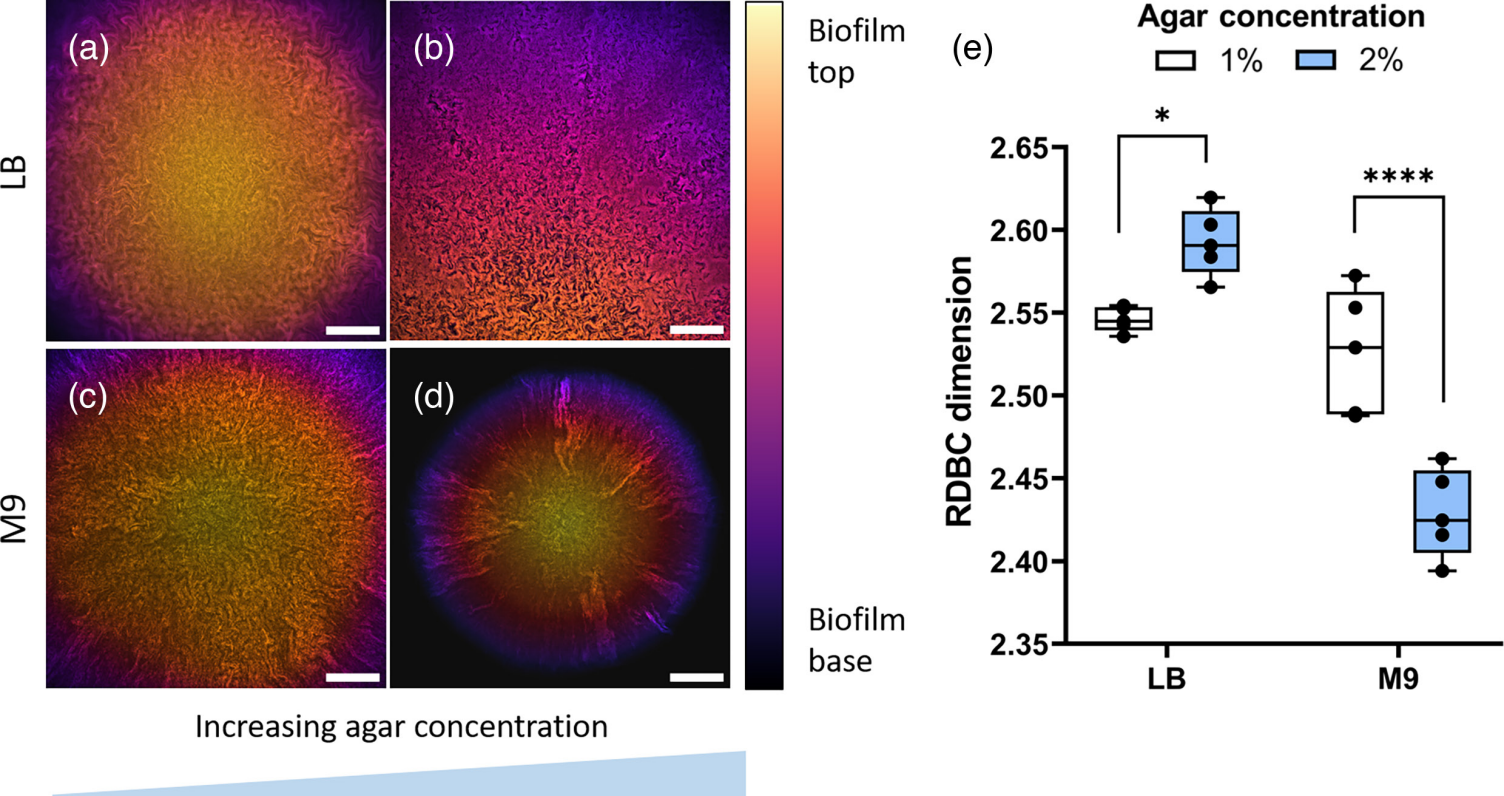
Morphology (a–d) and fractal complexity (e) of colony biofilms formed by the Δ*ompR* mutant strain grown on solid-rich (LB) and minimal (M9) substrates with agar concentrations of 1% (a, c) and 2% (b, d). The reduction in substrate stiffness leads to a decrease in complexity on the rich medium (*P* = 0.0117, *) and an increase in complexity on minimal medium (*P* = 2.39×10^−7^, ****). Average values are compared using one-way ANOVA tests. Colony biofilm images are projections of *z*-stacks acquired with 5 µm slice spacing, colour-coded by depth. *N* = 5 biofilms were imaged for each condition. Scale bars: 200 µm.

We further investigated the role of medium composition on Δ*ompR* biofilm internal morphology by comparing colony biofilms grown on LB and M9 substrates with different chemical compositions (Figs4  5). The RDBC dimension of colony biofilms grown in rich medium decreased considerably when the substrate was made softer by halving the amount of agar (*P* = 0.0117). On minimal medium, on the other hand, growth on soft (1% agar) substrates was associated with a significant increase in the RDBC dimension, comparable to that of biofilms grown on rich medium (*P* = 2.39×10^−7^).

**Fig. 5.**
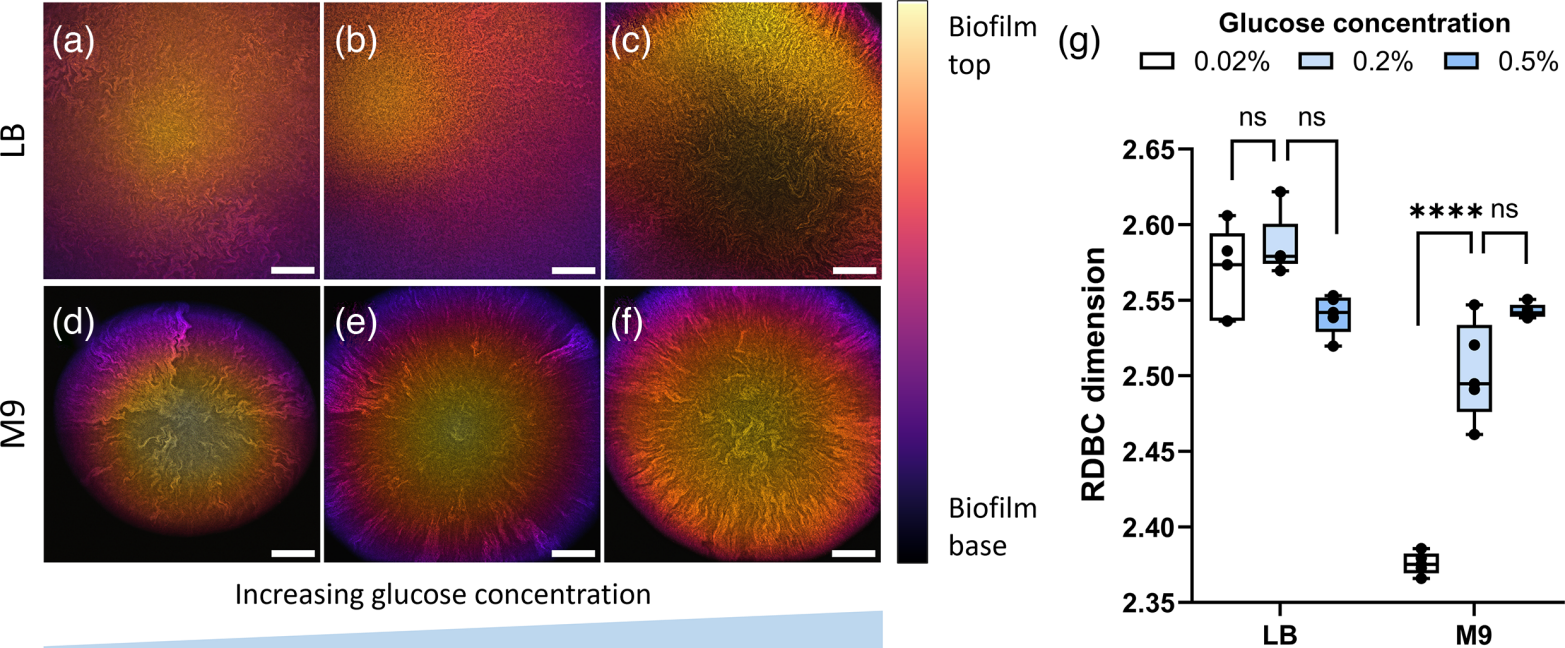
Morphology (a–f) and fractal complexity (g) of colony biofilms formed by the Δ*ompR* mutant strain grown on solid-rich (LB) and minimal (M9) substrates with glucose concentrations of 0.02% (a, d), 0.2% (b, e) and 0.5% (c, f). Glucose concentration in the medium is particularly important for the complexity of biofilms grown on minimal media: the RDBC dimension increases when glucose levels are increased from 0.02 to 0.2% (*P* = 2.78×10^−11^, ****). On rich medium, conversely, the addition of glucose does not significantly affect biofilm fractal complexity. Average values are compared using one-way ANOVA tests. Colony biofilm images are projections of *z*-stacks acquired with 5 µm slice spacing, colour-coded by depth. *N* = 5 biofilms were imaged for each condition. Scale bars: 200 µm.

Furthermore, increasing glucose amounts in minimal medium led to an increase in RDBC from 2.376 ± 0.007 (0.02% glucose) to 2.503 ± 0.032 (0.2 % glucose, *P* = 2.78×10^−11^) to 2.543 ± 0.005 (0.5 % glucose). In Δ*ompR*, this increase in the RDBC dimension coincided with the gradual disappearance of colony sectoring, brought by the increase in nutrient levels. As expected, the addition of the same amounts of glucose to rich medium did not lead to any significant changes in morphological complexity, with RDBC dimensions (2.520–2.622) comparable to those found in nominal rich medium with no glucose (2.566–2.620).

While the diameter of Δ*ompR* biofilms increased with glucose concentration and decreased with agar concentration, the presence of the dark background in images of smaller colony biofilms did not significantly affect the resulting RDBC dimension. This was checked by digitally zooming into images of the biofilms grown on 0.02% glucose, which had the smallest base area, until they filled the full field of view, and by successively re-calculating their RDBC dimension (Fig. S3). The resulting RDBC value was calculated as 2.373 ± 0.006, a decrease of only 0.1% compared to the original images. This difference in RDBC values is likely due to the digital rescaling of the zoomed-in image.

### Osmotic stress partially reduces complexity in Δ*ompR* colony biofilms

**Fig. 6.**
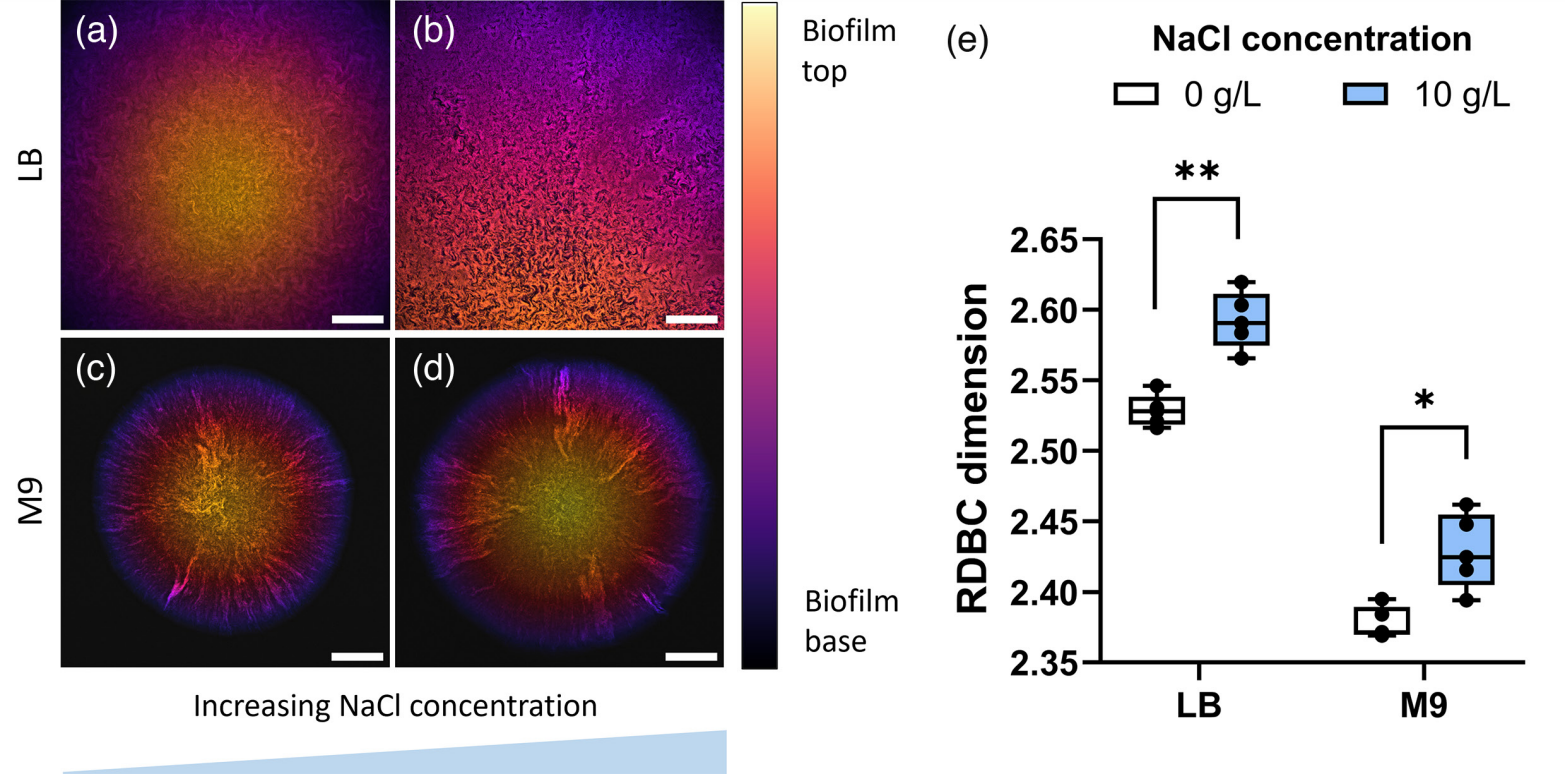
Morphology (a–d) and fractal complexity (e) of colony biofilms formed by the Δ*ompR* mutant strain grown in the solid-rich (LB) and minimal (M9) substrates with NaCl concentrations of 0 g l^−1^ (a, c) and 10 g l^−1^ (b, d). The presence of 10 g l^−1^ NaCl is associated with an increase in morphological complexity for both rich and minimal media (*P* = 7.78×10^−4^, ** and *P* = 0.0175, * respectively), as indicated by the RDBC dimension metric. Average values are compared using one-way ANOVA tests. Colony biofilm images are projections of *z*-stacks acquired with 5 µm slice spacing, colour-coded by depth. *N* = 5 biofilms were imaged for each condition. Scale bars: 200 µm.

Because of the role of *ompR* in osmotic stress response, the fractal complexity of colony biofilms formed by the mutant strain Δ*ompR* was studied on LB and M9/glucose media with and without salt (Fig. 6). A reduction in the RDBC dimension was observed when the osmolality of the medium was reduced by removing salt, both in rich (*P* = 7.78×10^−4^) and in minimal (*P* = 0.0175) media. This suggested that osmotic stress might control the morphology of colony biofilms formed by the Δ*ompR* mutant strain.

For this reason, the role of osmotic stress in the growth of the Δ*ompR* mutant strain was investigated at both the cellular and biofilm levels. This was achieved by measuring changes in cell length and width and by measuring the RDBC dimension of colony biofilm images grown in solid media with different osmolalities, respectively. Changes in growth medium osmolality were induced by adding different amounts of iodixanol, a mildly hypertonic compound originally designed for density gradient preparations [34], which can be used to perturb the osmotic pressure in the biofilm growth environment. This compound was chosen due to its inability to be metabolized by bacterial cells. This is in contrast with the dissociated Na^+^ and Cl^−^ ions from NaCl, whose assimilation can lead to physiological changes affecting strain phenotype and, consequently, channel fractality. Iodixanol toxicity in both the parental strain BW25113 and the Δ*ompR* mutant was checked by growth curve experiments (Fig. S4). At the cellular level, an increase in osmotic stress in the liquid growth media led to a reduction in cell size for both strains (Fig. S5). A significant reduction in cell length with increasing iodixanol concentration was observed in both strains: from 3.520 ± 0.827 µm to 2.205 ± 0.516 µm for BW25113 (*P* = 1.32×10^−167^) and from 3.878 ± 1.133 µm to 2.685 ± 0.657 µm for Δ*ompR* (*P* = 6.74×10^−40^). By contrast, cell width reduction with increasing iodixanol concentration was marginal for BW25113 (from 1.001 ± 0.099 µm to 0.970 ± 0.098 µm, *P* = 8.57×10^−8^), whereas it was almost five times higher for the Δ*ompR* strain (from 1.335 ± 0.219 µm to 1.147 ± 0.149 µm, *P* = 1.02×10^−13^).

**Fig. 7.**
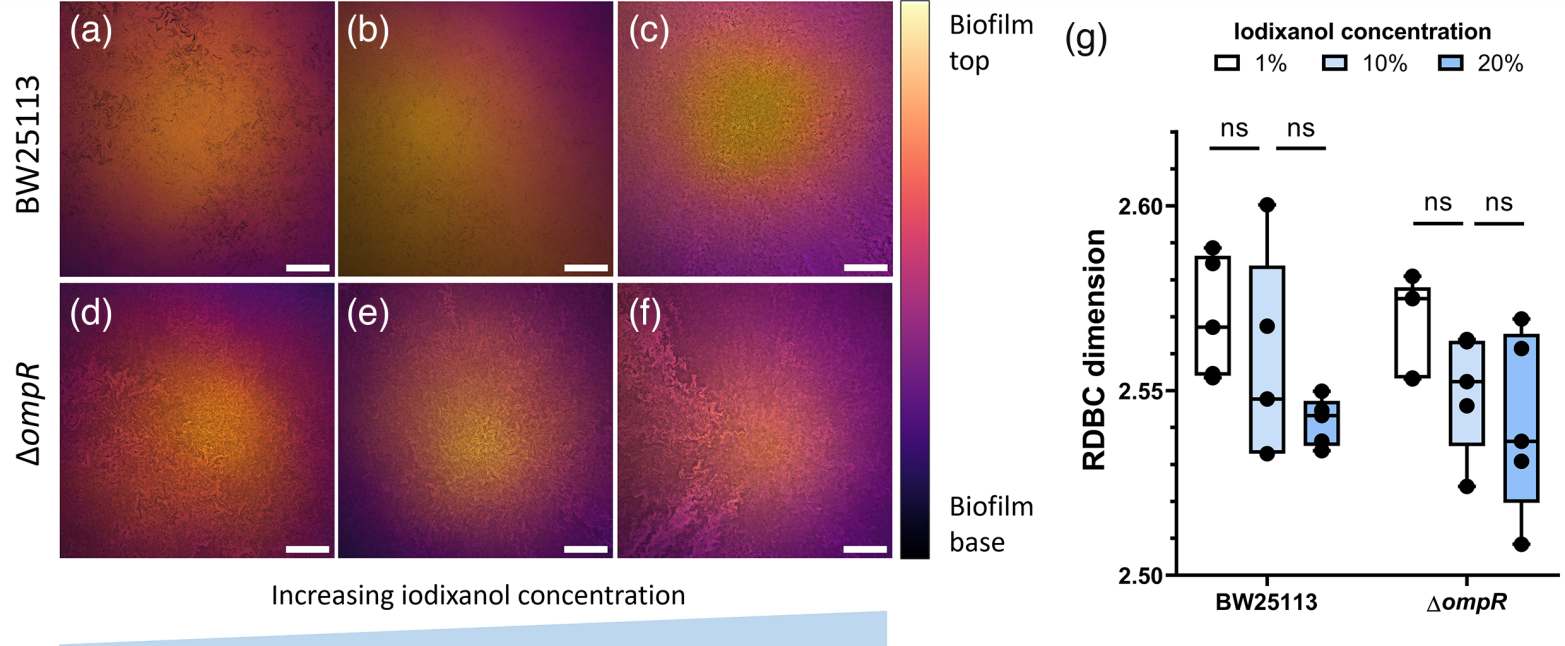
Comparison of fractal morphology between colony biofilms formed by the parental strain BW25113 and the mutant strain Δ*ompR* on solid substrates with increasing v/v iodixanol concentrations of 1% (a, d), 10% (b, e) and 20% (c, f). Colony biofilm images (a–f) are projections of *z*-stacks acquired with 5 µm slice spacing, colour-coded by depth. The average RDBC dimension decreases with increasing amounts of iodixanol and is similar between the two strains for each iodixanol concentration (g). *N* = 5 biofilms were imaged for each condition. Scale bars: 200 µm.

In colony biofilms, an increase in the iodixanol concentration in the solid growth substrate was associated with a small overall reduction in the RDBC dimension for both strains (Fig. 7); however, one-way ANOVA statistical tests comparing the mean RDBC values for each condition resulted non-significant. This loss of internal complexity could occur because planktonic growth is limited in high-osmolality medium (Fig. S4) or it could be a result of the cell size reduction following osmotic stress (Fig. S5). Alternatively, it could be a pleiotropic effect caused by the presence of iodixanol in the growth medium.

## Discussion

In this study, we present a systematic, quantitative characterization of *E. coli* intra-colony channel morphology by fractal image analysis of biofilm confocal micrographs. The extraction of fractal geometry parameters from biofilm micrographs (see, for example, [35]) is usually achieved by image thresholding, a type of image segmentation that isolates an object from its background depending on its greyscale value [36]. This process is straightforward in cases where the fractal feature to be analysed is fully contained in a separate imaging channel, such as in the fractal boundary between isogenic strains studied by Rudge *et al*. [11]. However, in biofilms formed by a single population of bacteria that express the same fluorescent protein, the binarization process is not effective for the detection of internal channel networks, where the difference in greyscale values between the channels and the rest of the biofilm is small. In our work, this obstacle is overcome using the fractal analysis software ComsystanJ. While this plugin cannot specifically extract channel networks from microscopy images, it calculates the RDBC dimension as a function of greyscale-level variation through space. The greatest variation in greyscale levels within the biofilm micrographs presented in this study is brought by the contrast between intra-colony channels and fluorescent cells. This justifies the use of ComsystanJ as a robust method for the calculation of the fractal complexity of internal channel networks. While a 3D version of the algorithm for calculating the RDBC fractal dimension exists in ComsystanJ, it presently only operates in binary mode. When applied to the greyscale microscopy images acquired in this study, the binarization process is automatic, but the binarization method is not specifically stated and the binary mask produced by the algorithm is not presented. For these reasons, the calculation of the RDBC fractal dimension was carried out using the 2D version of the algorithm on 2D maximum intensity projections of our biofilm image data. An expansion of the algorithm to allow for 3D greyscale images would allow more precise quantification of channel fractal complexity in the context of the biofilm volume.

Quantitative analysis of biofilm microscopy images revealed that the fractal morphology of *E. coli* nutrient transport channels is comparable to that of computer-generated fractals for all cell shape mutants and growth conditions investigated. While cell shape affects the overall biofilm morphology, this does not lead to a significant change in fractal complexity, as described by the RDBC dimension metric. This is likely a result of the genotype of the mutant strains investigated, which not only evoke an altered cell morphotype but can also affect other aspects of microbial physiology and metabolism. A wider panel of *E. coli* mutant morphotypes (such as spherical KJB24 cells [11] or the wide REL606*mreB* A53K cells [37]) could be used in conjunction with the image analysis methods presented in this work to precisely attribute changes in channel fractal morphology to cell shape phenotype.

We observed no statistically significant difference in the morphological complexity of channels formed by either the Δ*amiA* or Δ*ydgD* strains in this study. The deletion of the *N*-acetylmuramoyl-l-alanine amidase in BW25113 Δ*amiA*, which results in a long-cell phenotype, may preserve the spatial patterning of nutrient transport channels by maintaining the mechanical buckling forces that have previously been linked to the formation of linear biofilm structures, such as Van Gogh’s bundles and nematic ordering in chained-cell biofilms [38,40]. Our observation that deletion of the putative serine-type endopeptidase in BW25113 Δ*ydgD*, resulting in wide mutant cells, did not lead to the same altered fractality as the Δ*ompR* mutant further supports our hypothesis that the basic morphotype may not be the sole governing factor of channel complexity when comparing Δ*ydgD* and Δ*ompR*. However, the mechanistic role of YdgD pertaining to cell shape, metabolism and biofilm patterning remains unexplored in wider literature and demands further investigation to understand how channel patterning is preserved in these mutants.

In the case of the wide-cell mutant strain Δ*ompR*, fractal image analysis identified the role of nutrient availability and osmotic stress on the morphological complexity of channel networks. In particular, increasing glucose concentrations in minimal medium led to proportionally more complex biofilm channel organization, eventually reaching an RDBC dimension value close to that of biofilms grown on rich media. A similar effect was previously observed in *E. coli* JM105 mini-Tn7-*gfp* colony biofilms, which developed more complex channel patterns when grown on minimal medium with an excess of glucose compared to glucose-limited substrates [17]. Similarly, in this case, the increase in channel network complexity could allow for more efficient nutrient transport and access within the biofilm. Furthermore, we found that the growth on rich-soft substrates reduced biofilm morphological complexity, whereas growth on soft-minimal substrates increased it. This is in contrast with previous findings on *E. coli* JM105 mini-Tn7-*gfp*, where channels from biofilms grown on rich, soft substrates were densely packed [17]. We also investigated the morphology of Δ*ompR* cells and colony biofilms under growth conditions with varying osmolality owing to the role of OmpR in regulating the osmotic stress response in *E. coli* [41]. Gram-negative bacteria, such as *E. coli,* respond to an increase in external osmotic pressure by accumulating solutes inside the cell and by pumping out water through efflux [42]. Hyperosmotic shock also leads to a sudden cell volume shrinkage [43], followed by a gradual recovery [44], and is associated with a reduction in cell elongation rate [45]. NaCl-induced osmotic shock leads to a reduction in *E. coli* cell size in a process mediated by the morphogene *bolA* [46], which in turn is influenced by the stress response master regulator *rpoS* [47]. Low NaCl levels were associated with a lower fractal complexity on both rich and minimal media; however, NaCl affects not only medium osmolality but also nutrient metabolism [48]. Iodixanol, which cannot be metabolized by bacterial cells, was hence subsequently used to exclusively alter the osmolality of growth medium and produced the opposite effect on the RDBC dimension. This suggests that the metabolic properties of medium NaCl levels affected channel morphology more than their osmotic properties. Furthermore, the observed reduction in Δ*ompR* cell width in high-osmolality medium indicates a possible partial reversion to the parental phenotype, which could equally be driven by *ompR* suppressor mutations. This phenotypic reversion in cell shape may also explain the similarity in absolute RDBC dimension values between BW25113 and Δ*ompR* colony biofilms at each iodixanol concentration.

The use of fractal image analysis has proven to be a simple yet powerful method for the quantification of *E. coli* colony biofilm internal channels formed by cell shape mutants on different growth substrates. This method could be used on a wider panel of *E. coli* mutant strains to elucidate the mechanisms governing channel formation and development, or it could be readily applied to other microbial species exhibiting complex biofilm internal patterns.

## supplementary material

10.1099/mic.0.001511 Uncited Supplementary Material 1.

## Abbreviations

LB: lysogeny broth
RDBC: relative differential box counting
